# Evasion of Human Neutrophil-Mediated Host Defense during *Toxoplasma gondii* Infection

**DOI:** 10.1128/mBio.02027-17

**Published:** 2018-02-13

**Authors:** Tatiane S. Lima, Lanny Gov, Melissa B. Lodoen

**Affiliations:** a Department of Molecular Biology and Biochemistry, University of California, Irvine, Irvine, California, USA; b Institute for Immunology, University of California, Irvine, Irvine, California, USA; Albert Einstein College of Medicine

**Keywords:** IL-1, *Toxoplasma gondii*, immune evasion, inflammasome, neutrophils

## Abstract

Neutrophils are a major player in host immunity to infection; however, the mechanisms by which human neutrophils respond to the intracellular protozoan parasite *Toxoplasma gondii* are still poorly understood. In the current study, we found that, whereas primary human monocytes produced interleukin-1beta (IL-1β) in response to *T. gondii* infection, human neutrophils from the same blood donors did not. Moreover, *T. gondii* inhibited lipopolysaccharide (LPS)-induced IL-1β synthesis in human peripheral blood neutrophils. IL-1β suppression required active parasite invasion, since heat-killed or mycalolide B-treated parasites did not inhibit IL-1β release. By investigating the mechanisms involved in this process, we found that *T. gondii* infection of neutrophils treated with LPS resulted in reduced transcript levels of *IL-1β* and *NLRP3* and reduced protein levels of pro-IL-1β, mature IL-1β, and the inflammasome sensor NLRP3. In *T. gondii*-infected neutrophils stimulated with LPS, the levels of MyD88, TRAF6, IKKα, IKKβ, and phosphorylated IKKα/β were not affected. However, LPS-induced IκBα degradation and p65 phosphorylation were reduced in *T. gondii-*infected neutrophils, and degradation of IκBα was reversed by treatment with the proteasome inhibitor MG-132. Finally, we observed that *T. gondii* inhibited the cleavage and activity of caspase-1 in human neutrophils. These results indicate that *T. gondii* suppression of IL-1β involves a two-pronged strategy whereby *T. gondii* inhibits both NF-κB signaling and activation of the NLRP3 inflammasome. These findings represent a novel mechanism of *T. gondii* evasion of human neutrophil-mediated host defense by targeting the production of IL-1β.

## IMPORTANCE

*Toxoplasma gondii* is an obligate intracellular parasite that infects approximately one-third of humans worldwide and can invade virtually any nucleated cell in the human body. Although it is well documented that neutrophils infiltrate the site of acute *T. gondii* infection, there is limited understanding of how human neutrophils respond to *T. gondii*. Neutrophils control infectious pathogens by a variety of mechanisms, including the release of the cytokine IL-1β, a major driver of inflammation during infection. This study reveals that *T. gondii* is able to inhibit IL-1β production in human neutrophils by impairing the activation of the NF-κB signaling pathway and by inhibiting the inflammasome, the protein complex responsible for IL-1β maturation. This two-pronged strategy of targeting the IL-1β pathway may facilitate the survival and spread of *T. gondii* during acute infection.

## INTRODUCTION

Neutrophils control infectious pathogens by phagocytosing and degrading microbes, releasing granules with lytic enzymes, and producing reactive oxygen species (ROS) (1, 2). Neutrophils can also contain and eliminate extracellular microbes by releasing neutrophil extracellular traps (NETs) (3). More recently, it has been shown that neutrophil functions extend beyond these roles in acute infection. It is now appreciated that they are antimicrobial effectors that can also shape inflammatory responses through the release of chemokines and cytokines (1) and that they can contribute to adaptive immunity through cross-talk with other cell types, such as macrophages, dendritic cells, and lymphocytes (4).

*Toxoplasma gondii* is an obligate intracellular parasite that infects an estimated one-third of the global human population (5). Human exposure to *T. gondii* typically occurs due to ingestion of parasite cysts in contaminated food or water (6), but it can also occur due to vertical transmission from mother to fetus (7). As an oral pathogen, *T. gondii* enters the body and establishes infection in the small intestine (5). The parasite then disseminates via the bloodstream and surmounts a variety of biological barriers to establish chronic infection in several different organs, including the heart and brain (8).

Neutrophils are rapidly recruited to sites of *T. gondii* infection in mice (9, 10). Although neutrophils possess an arsenal of antimicrobial effector mechanisms, remarkably, *T. gondii* can survive and replicate in mouse and human neutrophils, and in *T. gondii*-infected mice, neutrophils in the small intestine and lymph nodes contain replicating parasites (11–14). Neutrophils produce interleukin-12 (IL-12), tumor necrosis factor alpha (TNF-α), interferon gamma (IFN-γ), and NETs in response to *T. gondii* (15–17) and contribute to dendritic cell activation (18). Despite these host-protective functions for neutrophils, the specific depletion of neutrophils with anti-Ly6G monoclonal antibodies (MAb) resulted in only a slight increase in mortality compared with high mortality in mice depleted of both monocytes and neutrophils using the anti-Ly6C/G MAb RB6-8C5 (19), suggesting that monocytes may play a more critical role in immune defense against *T. gondii* (20). One potential explanation for these data is that despite the recruitment of neutrophils to sites of infection, *T. gondii* is able to evade the neutrophil immune response. Most studies on neutrophil immunity during *T. gondii* infection have focused on infection in mice. In contrast, little is known at the molecular level about the interactions of *T. gondii* with human neutrophils.

Interleukin-1beta (IL-1β) is a key regulator of inflammation that activates a variety of downstream inflammatory genes (21). *T. gondii* induces IL-1β in multiple human primary cells and cell lineages, and *T. gondii*-induced IL-1β mediates host protection against the parasite (22–26). The production of IL-1β is regulated by the inflammasome, a multiprotein complex typically composed of caspase-1, an adaptor protein ASC (apoptosis-associated speck-like protein), and a cytosolic sensor, which can be either a nucleotide oligomerization domain (NOD)-like receptor (NLR) or an AIM2-like receptor (ALR) (27). The best-studied inflammasome is the NLRP3 inflammasome, which can be activated by a wide variety of stimuli, including ATP, bacterial toxins, microbial products, endogenous molecules, and particulate matter (28–30). A two-signal model for NLRP3 inflammasome activation in macrophages has emerged. Stimulation of receptors that induce NF-κB signaling leads to transcriptional upregulation of *IL-1β* and *NLRP3* (31). A second signal, such as ATP, activates the inflammasome to proteolytically process pro-IL-1β into mature, bioactive IL-1β, which is released by the cell. Neutrophils also produce IL-1β in infection and inflammatory diseases (32–34) and use this two-signal model for inflammasome activation (35). In addition, a one-signal mechanism that requires only lipopolysaccharide (LPS) to activate both NF-κB signaling and the inflammasome in neutrophils and monocytes has been described (36–38).

We have previously demonstrated that *T. gondii* triggers IL-1β release from human monocytes via a pathway dependent on NLRP3, ASC, caspase-1, and K^+^ efflux (26, 39). Here, we report that unlike in monocytes, *T. gondii* does not induce IL-1β in primary human neutrophils and actually inhibits LPS-induced IL-1β production in these cells. *T. gondii*-induced IL-1β suppression is associated with a reduction in NF-κB activation and *IL-1β* and *NLRP3* transcripts and a lack of activation of the NLRP3 inflammasome. These data indicate a novel two-pronged strategy of immune evasion in which *T. gondii* downregulates the inflammatory response of human neutrophils. This strategy may facilitate the survival and spread of the parasite during acute infection, particularly in the gut, where neutrophils encounter both *T. gondii* and Gram-negative bacteria that contain LPS.

## RESULTS

### *T. gondii* infects and survives in primary human neutrophils.

**FIG 1:**
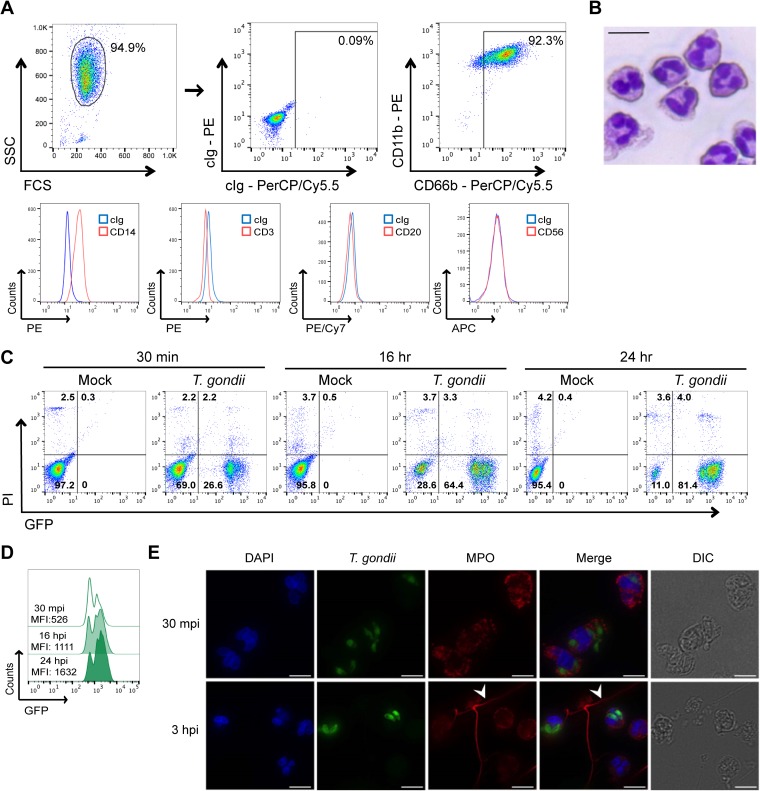
Isolation and infection of human peripheral blood neutrophils with GFP-expressing *T. gondii*. Neutrophils were isolated from human peripheral blood through density gradients. (A) Cells were stained with control Ig or anti-CD66b, anti-CD11b, anti-CD14, anti-CD3, anti-CD20, or anti-CD56, and flow cytometry was performed. The results of a representative analysis are shown. FCS, forward scatter; SSC, side scatter; cIg, control immunoglobulin. (B) Cells were stained with May-Grünwald-Giemsa solution and analyzed by light microscopy (40×). Bar, 15 µm. (C) Neutrophils were infected with type I *T. gondii* (RH strain) and stained with propidium iodide (PI), and at 30 min postinfection (mpi) and 16 and 24 h postinfection (hpi), the infection efficiency and cell viability were measured by flow cytometry. (D) The mean fluorescence intensity (MFI) of the GFP^+^ (infected) cells was determined at 30 mpi and at 16 and 24 hpi. (E) *T. gondii*-infected neutrophils were fixed, permeabilized, stained with an antibody against myeloperoxidase (MPO), counterstained with DAPI, and examined by immunofluorescence microscopy (60×). The white arrowheads indicate neutrophil extracellular traps (NETs). Bars, 10 µm. These experiments were performed four (A) and three (B, C, D, and E) times with different donors. The results of representative experiments are shown. DIC, differential interference contrast.

Primary human neutrophils were isolated from peripheral blood samples drawn from healthy donors and separated through a density gradient. The isolated population was analyzed by flow cytometry, and 92% of the cells were confirmed to be neutrophils, as they were CD66b^+^ CD11b^+^ CD14^low^ and negative for T (CD3), B (CD20), and NK (CD56) cell markers (Fig. 1A). Analysis of the cells by microscopy confirmed that the population consisted predominantly of neutrophils due to the characteristic multilobed nuclei and the acidophilic cytoplasm with small granules (Fig. 1B). Isolated neutrophils were infected with green fluorescent protein (GFP)-expressing *T. gondii*, and the infection efficiency and cell viability were analyzed over time. These conditions resulted in approximately 26% infection efficiency at 30 min postinfection (mpi), 64% at 16 h postinfection (hpi), and 81% at 24 hpi (Fig. 1C). Moreover, the mean fluorescence intensity (MFI) of the GFP-positive (GFP^+^) population increased over time, indicating that *T. gondii* could survive inside human neutrophils and that the parasite burden increased (Fig. 1D), either due to replication or to multiple invasion events. Immunofluorescence assays of infected neutrophils at 30 mpi and 3 hpi confirmed the presence of multiple intracellular parasites and also demonstrated the release of extracellular traps in response to *T. gondii* (Fig. 1E), as has been previously published (17). The cell viability was greater than 90% in unstimulated, *T. gondii*-infected, and LPS-treated neutrophils cultivated in the presence of 10% fetal bovine serum (FBS) (Fig. 1C; also see Fig. S1 in the supplemental material).

10.1128/mBio.02027-17.1 FIG S1  Cell viability of unstimulated, *T. gondii*-infected, and LPS-treated neutrophils. Neutrophils were treated as follows: (i) mock infected, (ii) infected with GFP-expressing type I *T. gondii*, (iii) infected and stimulated with LPS (500 ng/ml), or (iv) stimulated with LPS only for 16 h. Infection efficiency and cell viability, based on PI staining, were measured by flow cytometry. Download FIG S1, PDF file, 0.2 MB. Copyright © 2018 Lima et al. 2018 Lima et al. This content is distributed under the terms of the Creative Commons Attribution 4.0 International license.

### *T. gondii* infection inhibits LPS-induced IL-1β in primary human neutrophils.

**FIG 2:**
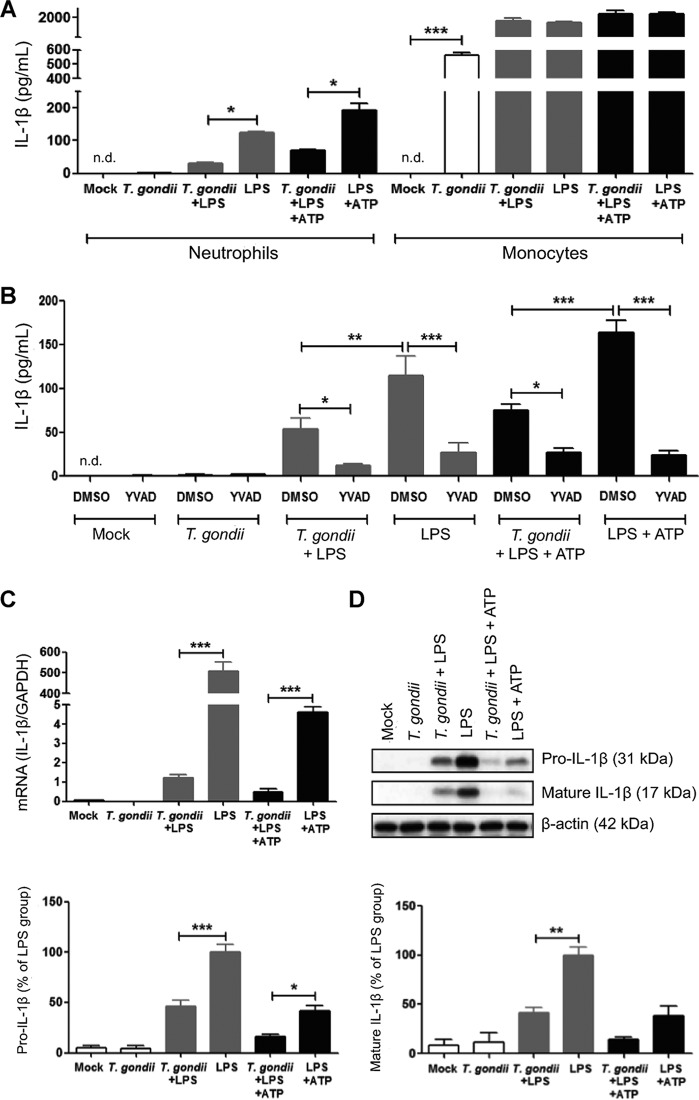
IL-1β synthesis is inhibited in *T. gondii*-infected primary human neutrophils. Neutrophils and monocytes isolated from the same donors were treated as follows: (i) mock infected, (ii) infected with *T. gondii*, (iii) infected with *T. gondii* and stimulated with LPS (500 ng/ml), (iv) stimulated with LPS only (positive control), (v) infected and stimulated with LPS+ATP (5 mM), or (vi) stimulated with LPS+ATP only (positive control). (A) After 16 h, IL-1β released into the culture supernatant was measured by ELISA. (B) Neutrophils were treated with either DMSO (vehicle control) or 100 µM Ac-YVAD-CMK (YVAD), a specific caspase-1 inhibitor for 30 min before infection, and at 16 hpi, IL-1β released into the culture supernatant was measured by ELISA. (C) Q-PCR was performed with specific primers for IL-1β or GAPDH. The transcript levels relative to those of GAPDH from a representative experiment are shown. (D) Pro-IL-1β and mature IL-1β in the cell lysate were visualized and quantified by Western blotting. Data are combined from 3 (A, C, and D) and 9 (B) experiments with different donors. Values are expressed as the means plus standard errors of the means (SEM) (error bars). Values that are significantly different are indicated by a bar and asterisk as follows: *, *P* < 0.05; **, *P* < 0.01; ***, *P* < 0.001 (one-way ANOVA followed by a Bonferroni posttest in panel A and a Tukey posttest in panels B, C, and D). n.d., not detected.

Given the large number of neutrophils recruited to sites of acute *T. gondii* infection (11, 14, 40) and the importance of IL-1β in immunity and inflammation during infection (41), we evaluated the secretion of IL-1β into the culture supernatant of infected human neutrophils. Since two different pathways can lead to IL-1β release by neutrophils (involving either one or two signals), for positive controls, we stimulated neutrophils with LPS or with LPS+ATP (35, 38). We observed that primary human monocytes produced IL-1β in response to *T. gondii* infection, as previously shown (39), whereas human neutrophils from the same donors did not (Fig. 2A). Surprisingly, in the presence of LPS or LPS+ATP, *T. gondii* infection actually inhibited IL-1β release by neutrophils (Fig. 2A). To confirm a role for the protease, caspase-1, in the production of IL-1β in human neutrophils, we used Ac-YVAD-CMK, a cell-permeable tetrapeptide inhibitor that binds to the active site of caspase-1 and prevents substrate interaction (42). We confirmed by flow cytometry that the caspase-1 inhibitor did not affect the infection efficiency or cell viability compared to the vehicle control (Fig. S2). However, IL-1β release was significantly reduced in the presence of the caspase-1 inhibitor compared to the dimethyl sulfoxide (DMSO) control, and this reduction was observed in both positive controls (LPS and LPS+ATP) and also in cells that were stimulated with LPS or LPS+ATP and infected with *T. gondii* (Fig. 2B).

10.1128/mBio.02027-17.2 FIG S2  Infection efficiency and viability of neutrophils treated with DMSO or YVAD. Neutrophils were pretreated with either DMSO (vehicle control) or 100 µM Ac-YVAD-CMK (YVAD), a specific caspase-1 inhibitor for 30 min. Neutrophils were then infected, and after 16 h, infection efficiency and cell viability were measured by flow cytometry. Download FIG S2, PDF file, 0.2 MB. Copyright © 2018 Lima et al. 2018 Lima et al. This content is distributed under the terms of the Creative Commons Attribution 4.0 International license.

We next examined whether *T. gondii* inhibited only the secretion of IL-1β or also affected IL-1β synthesis. Quantitative real-time PCR (Q-PCR) analysis revealed that *T. gondii* infection strongly inhibited LPS-induced *IL-1β* transcript levels (Fig. 2C). Because IL-1β is synthesized as a zymogen, pro-IL-1β, which is posttranslationally processed into mature IL-1β (43), we investigated IL-1β processing in the lysate of infected and/or stimulated neutrophils by Western blotting. We were able to analyze the lysates, but not the supernatants, of neutrophils by Western blotting, since analysis of the supernatant requires culturing the cells in serum-free medium, which neutrophils do not tolerate. By analyzing the lysates, we found that mock-infected and *T. gondii*-infected neutrophils did not harbor a pool of pro- or mature IL-1β; however, we detected pro- and mature IL-1β in neutrophils treated with LPS or LPS+ATP (Fig. 2D). Both forms of IL-1β were more abundant in the lysates of LPS than in the lysates of LPS+ATP-treated neutrophils, likely because LPS+ATP consistently resulted in greater release of IL-1β into the supernatants. In contrast, *T. gondii* infection reduced the levels of pro- and mature IL-1β in LPS- or LPS+ATP-treated samples (Fig. 2D), suggesting that *T. gondii* inhibited LPS-induced synthesis of IL-1β, which resulted in reduced IL-1β release from *T. gondii*-infected neutrophils.

### Inhibition of LPS-induced IL-1β by *T. gondii* infection requires active parasite invasion.

**FIG 3:**
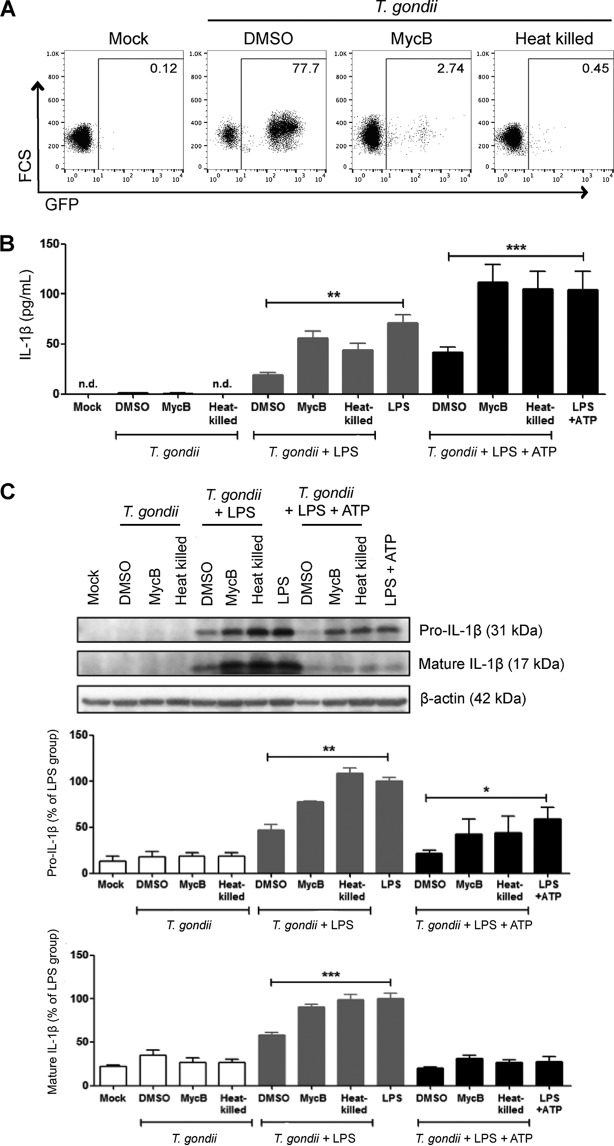
Effect of blocking parasite invasion on IL-1β inhibition. GFP-expressing *T. gondii* were heat killed, treated with DMSO (vehicle control), or pretreated with 5 µM mycalolide B (MycB) and added to unstimulated or stimulated neutrophils (stimulated with LPS or LPS+ATP) for 16 h. Mock-infected cells were cultured in parallel. (A) Infection efficiency was measured by flow cytometry. (B) The amount of IL-1β released into the culture supernatant was measured by ELISA. (C) Pro-IL-1β and mature IL-1β in the cell lysate were visualized by Western blotting. Data reflect combined results of eight (A and B) and three (C) experiments with different donors. Values are expressed as the means plus SEM. Values that are significantly different by one-way ANOVA followed by a Bonferroni posttest are indicated by a bar and asterisk as follows: *, *P* < 0.05; **, *P* < 0.01; ***, *P* < 0.001. The results of representative Western blots are shown in panels A and C.

To test whether IL-1β inhibition required active parasite invasion, we cultured neutrophils with *T. gondii* parasites that were heat killed, pretreated with a vehicle control (DMSO), or pretreated with mycalolide B, an irreversible inhibitor of actin polymerization (44). Parasites treated with mycalolide B attach to host cells and secrete the contents of the rhoptries, a specialized secretory organelle of the parasite, but cannot invade, since invasion is powered by the parasite’s actin-myosin machinery. Importantly, *T. gondii* parasites were washed before addition to the neutrophils, so the mycalolide B did not affect the neutrophil actin cytoskeleton. When neutrophils were infected with DMSO-treated parasites for 16 h, approximately 70% of the cells were infected (GFP^+^). As expected, heat-killed parasites did not infect the neutrophils, and when parasites were pretreated with mycalolide B, <3% of the cells were infected (GFP^+^), indicating that both treatments effectively impaired invasion (Fig. 3A). Interestingly, when neutrophils were infected with heat-killed or mycalolide B-treated parasites, LPS-induced IL-1β synthesis and release into the culture supernatant were not inhibited compared to the control DMSO-treated parasites (Fig. 3B and C). These data indicate that *T. gondii*-mediated IL-1β inhibition required active parasite invasion and that host sensing of the parasite, parasite attachment, and rhoptry protein secretion alone were not sufficient for inhibiting IL-1β release.

### *T. gondii* inhibits activation of the NLRP3 inflammasome.

**FIG 4:**
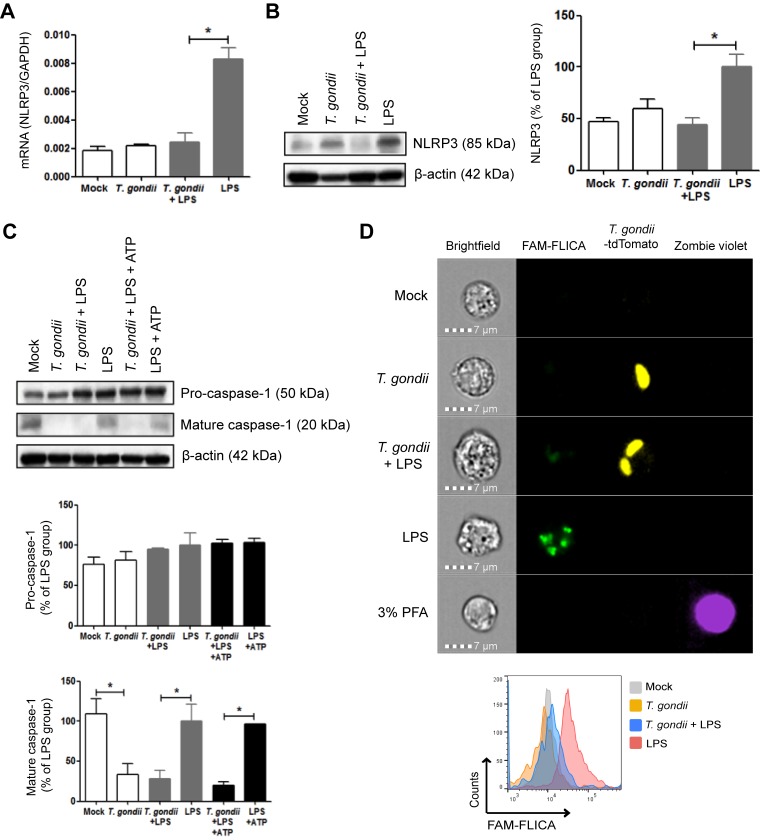
Effect of *T. gondii* infection on the NLRP3 inflammasome and its activation. Neutrophils were treated as follows: (i) mock infected, (ii) infected with *T. gondii*, (iii) infected with *T. gondii* and stimulated with LPS (500 ng/ml), (iv) stimulated with LPS only (positive control), (v) infected and stimulated with LPS+ATP (5 mM), or (vi) stimulated with LPS+ATP only (positive control). (A) After 16 h, Q-PCR was performed with specific primers for NLRP3 or GAPDH. The transcript levels relative to those of GAPDH are shown. (B and C) NLRP3 (B), pro-caspase-1 and cleaved caspase-1 (C) in the cell lysate were visualized and quantified by Western blotting. (D) Caspase-1 activity was detected by the FAM-FLICA probe and visualized and quantified by ImageStream flow cytometry. Zombie violet was used to assay for cell death, and the treatment of cells with 3% PFA for 30 min was used as a positive control for cell death. The histograms depict the levels of FLICA (caspase-1 enzymatic activity) in each population of cells. Each experiment was performed three times with different donors. Values are expressed as the means plus SEM. Values that are significantly different (*P* < 0.05 by one-way ANOVA followed by Tukey posttest) are indicated by a bar and asterisk. Representative blots are shown in panels B and C, and representative images are shown in panel D.

The NLRP3 inflammasome mediates IL-1β cleavage and release in neutrophils stimulated with LPS and ATP (35). Human and mouse peripheral blood neutrophils constitutively express NLRP3 protein, and its expression increases following Toll-like receptor (TLR) stimulation (35, 45). We first analyzed *NLRP3* transcript levels by Q-PCR. Mock-infected and *T. gondii*-infected neutrophils showed low levels of *NLRP3* transcripts, which were upregulated by LPS. In contrast, *T. gondii* infection reduced LPS-induced *NLRP3* transcripts (Fig. 4A). In addition, low levels of NLRP3 protein were confirmed by Western blotting of lysates from mock- and *T. gondii*-infected neutrophils, and in some donors, *T. gondii* infection induced NLRP3 protein expression to some extent. NLRP3 was consistently upregulated after LPS stimulation, and this upregulation was inhibited in cells that had been infected with *T. gondii* (Fig. 4B). Although the NLRP3 inflammasome can be activated differently due to stimulation with LPS or LPS+ATP (35, 37, 38, 46), a common step is the cleavage and activation of the protease caspase-1. To investigate the processing of caspase-1, we examined pro- and cleaved caspase-1 by Western blotting. Pro-caspase-1 levels were relatively constant among all the experimental groups. Mature caspase-1 was observed in unstimulated neutrophils, as previously reported (47), and these levels were not increased by LPS or LPS+ATP treatment; however, *T. gondii* infection alone or in the presence of LPS or LPS+ATP strongly inhibited caspase-1 cleavage (Fig. 4C). To directly evaluate caspase-1 enzymatic activity under each condition, we used the FAM-FLICA (ImmunoChemistry Technologies) probe on neutrophils treated with LPS and/or infected with tdTomato-expressing *T. gondii* and analyzed the cells by ImageStream flow cytometry. Although unstimulated neutrophils harbored cleaved caspase-1 based on Western blots, little to no caspase-1 activity was observed in these cells (Fig. 4D). The treatment of neutrophils with LPS induced high levels of caspase-1 activity, as shown by the numerous and intense FLICA specks (green). *T. gondii* infection alone did not induce caspase-1 activity, but parasite infection reduced LPS-induced caspase-1 activity, and greater than 95% of LPS-treated, infected cells were FLICA negative (Fig. 4D). In all experimental conditions, the cell viability was high, based on Zombie violet staining (3% paraformaldehyde [PFA] was used as a positive control for cell death). Collectively, these data demonstrate that *T. gondii* infection reduced LPS-induced NLRP3 transcript and protein expression and the processing and enzymatic activity of caspase-1.

### *T. gondii* infection inhibits LPS-induced NF-κB activation by reducing IκBα degradation.

**FIG 5:**
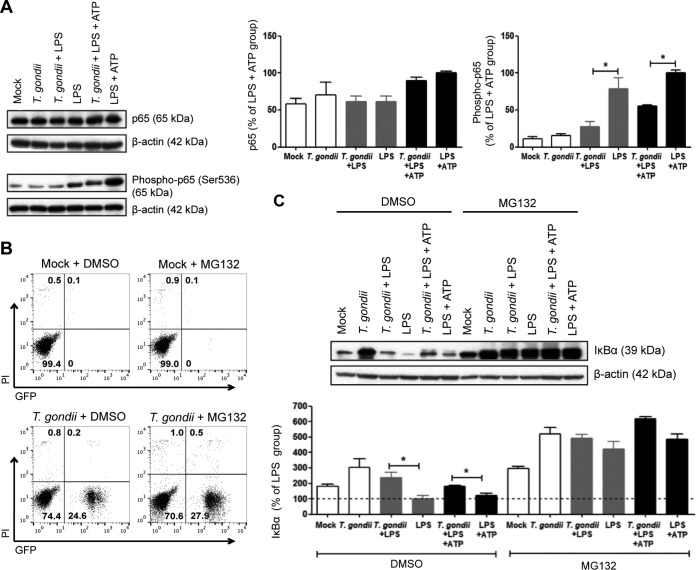
Effect of *T. gondii* infection on NF-κB activation in human neutrophils. Neutrophils were pretreated with either DMSO (vehicle control) or 50 µM MG-132, an inhibitor of the proteasome, for 1 h. Neutrophils were then treated as follows: (i) mock infected, (ii) infected with GFP-expressing type I *T. gondii*, (iii) infected and stimulated with LPS (500 ng/ml), (iv) stimulated with LPS only (positive control), (v) infected and stimulated with LPS+ATP (5 mM), or (vi) stimulated with LPS+ATP only (positive control) for 30 min. (A) Total p65 and phospho-p65 (Ser536) in the cell lysate were visualized and quantified by Western blotting. (B) Infection efficiency and cell viability were measured by flow cytometry. (C) IκBα in the cell lysate was visualized and quantified by Western blotting. Each experiment was performed three (A and B) or four (C) times with different donors. Values are expressed as the means plus SEM. Values that are significantly different (*P* < 0.05 by one-way ANOVA followed by Tukey posttest in panel A and a Bonferroni posttest in panel C) are indicated by a bar and asterisk. The results of representative experiments are shown in panels A, B, and C.

Since IL-1β and NLRP3 transcription are activated downstream of NF-κB signaling (31, 48) and *T. gondii* (RH strain) can suppress NF-κB activation in macrophages and fibroblasts (49–51), we hypothesized that *T. gondii* may inhibit IL-1β in human neutrophils via suppression of NF-κB signaling. Stimulation of cells with LPS leads to canonical NF-κB activation, which involves the phosphorylation and nuclear translocation of the NF-κB subunit p65 (52). We evaluated the levels of total and phospho-p65 in neutrophils 30 min after LPS treatment and/or *T. gondii* infection. Total p65 was comparable across all experimental groups; however, the stimulation of neutrophils with LPS or LPS+ATP strongly induced p65 phosphorylation (Fig. 5A), indicating activation of NF-κB signaling. In contrast, *T. gondii* infection reduced LPS-induced p65 phosphorylation, suggesting that *T. gondii* inhibited NF-κB signaling in human neutrophils. As expected, low levels of phospho-p65 were observed in mock-infected and *T. gondii*-infected cells (Fig. 5A).

On the basis of these findings, we investigated the specific step of the NF-κB pathway that was inhibited by *T. gondii* infection. To do this, we analyzed MyD88, TRAF6, IKKα, IKKβ, and phospho-IKKα/β (p-IKKα/β), which we found were not affected by infection (Fig. S3), and LPS-induced IκBα degradation (Fig. 5C). IκB proteins are cytoplasmic inhibitors of NF-κB that are phosphorylated, ubiquitinated, and degraded to allow nuclear translocation of NF-κB dimers and activation of transcription (53). Reduced levels of IκBα were observed in neutrophils stimulated with LPS or LPS+ATP compared to mock-infected or *T. gondii*-infected neutrophils (Fig. 5C). In contrast, in cells that were both infected and stimulated with LPS or LPS+ATP, IκBα was not degraded to the same degree as in uninfected cells stimulated with LPS or LPS+ATP, indicating that *T. gondii* infection inhibited LPS-induced IκBα degradation. Moreover, *T. gondii* infection alone inhibited the basal turnover of IκBα in neutrophils (Fig. 5C). To confirm degradation of IκBα by the proteasome, we used the proteasome inhibitor MG-132 (54), which reversed IκBα degradation in all samples, demonstrating that LPS-induced IκBα degradation was dependent on the proteasome (Fig. 5C). We confirmed by flow cytometry that the proteasome inhibitor did not affect the infection efficiency or cell viability compared to the vehicle control (Fig. 5B). Taken together, these findings demonstrate that *T. gondii* infection inhibited LPS-induced NF-κB activation in human neutrophils by reducing IκBα degradation and p65 nuclear translocation.

10.1128/mBio.02027-17.3 FIG S3  Effect of *T. gondii* infection on the early stages of NF-κB activation in human neutrophils. Neutrophils were treated as follows: (i) mock infected, (ii) infected with GFP-expressing type I *T. gondii*, (iii) infected and stimulated with LPS (500 ng/ml), (iv) stimulated with LPS only (positive control), (v) infected and stimulated with LPS+ATP (5 mM) or (vi) stimulated with LPS+ATP only (positive control) for 30 min. MyD88, TRAF6, IKKα, IKKβ, and phospho-IKKα/β in the cell lysate were visualized and quantified by Western blotting. Each experiment was performed three independent times. Values are expressed as the means plus SEM. Values that are significantly different (*P* < 0.05 by ANOVA followed by Tukey’s test) are indicated by a bar and asterisk. The results of representative Western blots are shown. Download FIG S3, PDF file, 0.5 MB. Copyright © 2018 Lima et al. 2018 Lima et al. This content is distributed under the terms of the Creative Commons Attribution 4.0 International license.

Although NF-κB is the main transcriptional activator of IL-1β, it has been demonstrated in human neutrophils that CREB1 and C/EBPβ activation are also involved in the induction of inflammatory cytokine genes (55, 56). Therefore, to determine whether *T. gondii*-mediated IL-1β inhibition was specifically associated with suppression of NF-κB signaling, we evaluated a possible effect of *T. gondii* infection in CREB-1 and C/EBPβ activation. We investigated the levels of total and phospho-CREB1 and total and phospho-C/EBPβ (both activator, LAP, and inhibitor, LIP isoforms) in neutrophils 30 min after LPS treatments and/or *T. gondii* infection. The levels of CREB-1 and C/EBPβ were comparable across all experimental groups (Fig. S4), indicating that *T. gondii*-mediated IL-1β inhibition in human neutrophils was not associated with regulation of these transcriptional factors but specifically with inhibition of the NF-κB pathway.

10.1128/mBio.02027-17.4 FIG S4  Effect of *T. gondii* infection on CREB-1 and C/EBPβ activation in human neutrophils. Neutrophils were treated as follows: (i) mock infected, (ii) infected with GFP-expressing type I *T. gondii*, (iii) infected and stimulated with LPS (500 ng/ml), (iv) stimulated with LPS only (positive control), (v) infected and stimulated with LPS+ATP (5 mM), or (vi) stimulated with LPS+ATP only (positive control) for 30 min. CREB-1, phospho-CREB-1 (Ser133) (A), C/EBPβ, and phospho-C/EBPβ (Thr188 and Thr37) (B) in the cell lysate were visualized and quantified by Western blotting. Each experiment was performed three independent times. Values are expressed as the means plus SEM. Values were compared by ANOVA followed by Tukey’s test. The results of representative experiments are shown in panels A and B. Download FIG S4, PDF file, 0.6 MB. Copyright © 2018 Lima et al. 2018 Lima et al. This content is distributed under the terms of the Creative Commons Attribution 4.0 International license.

## DISCUSSION

Neutrophils play a key role in acute inflammation and are rapidly recruited to sites of infection (1, 2). Neutrophils were identified by Ehrlich in 1880 (57), and their function as immune cells that migrate to sites of injury to digest microbes was observed in starfish embryos 13 years later by Metchnikoff (58). Remarkably, Metchnikoff’s findings, over 120 years later, still describe the basic role of neutrophils in innate immunity.

Although IL-1β is considered to be a product predominantly of monocytes and macrophages, neutrophils are also capable of producing this cytokine, and the large number of neutrophils found at sites of inflammation may compensate for the low levels of IL-1β synthesized per cell, resulting in a significant overall contribution (32, 59). Unlike in macrophages, IL-1β processing is not well understood in neutrophils. Mouse and human neutrophils can produce IL-1β through the canonical pathway involving two signals. In addition, neutrophils can secrete IL-1β in response to LPS alone, via a pathway that requires caspase-1 (60), Syk, ROS production, and lysosomal destabilization (38). Our data show a novel strategy of immune evasion in which *T. gondii* inhibits IL-1β production induced by LPS or LPS+ATP in primary human neutrophils. This mechanism may be particularly relevant during *T. gondii* infection in the small intestine. Since the human gut harbors a large number of commensal bacteria, LPS from gut microbes may shape the immune responses in this environment (61). Moreover, *T. gondii* infection is associated with cell damage and the release of danger-associated molecular patterns, such as ATP (62). Extracellular ATP leads to paracrine or autocrine activation of downstream purinergic signaling and exacerbation of the inflammatory response (63, 64).

Studies using rodents identified NLRP1 and NLRP3 as sensors for *T. gondii* (65–67) and show that mice deficient in either of these sensors display increased parasite burden and acute mortality (67). Recently, our group identified NLRP3 as a sensor for *T. gondii* in primary human monocytes (39). NLRP3 is expressed in mouse (45) and human (68) neutrophils and increases in response to stimulation with TLR ligands, including LPS (45, 68). However, we have found that *T. gondii* decreases LPS-induced NLRP3 expression in neutrophils, thereby hampering IL-1β production. Several pathogens have developed mechanisms to avoid or suppress inflammasome activation (69), but to our knowledge, the current report is the first evidence of a mechanism that relies, at least in part, on transcriptional inhibition of an inflammasome sensor.

In neutrophils, TLR agonists induce transcription of IL-1β and components of the inflammasome through NF-κB activation (48, 70). Interestingly, *T. gondii* can modulate the NF-κB pathway in different ways depending on the parasite strain. The dense granule protein GRA15 from type II *T. gondii* is an inducer of sustained NF-κB activation (71). In contrast, type I *T. gondii* induces IκB degradation but fails to induce p65 nuclear translocation in fibroblasts and macrophages (49, 51). Type I *T. gondii* also impairs the ability of LPS to activate NF-κB and to induce IL-12 and TNF-α in macrophages (49, 50). In neutrophils, our data similarly show that type I *T. gondii* did not activate NF-κB but inhibited LPS-induced NF-κB signaling at the level of inhibition of IκB degradation. Since we did not detect an effect of *T. gondii* on several of the proteins upstream of IκB (see Fig. S2 in the supplemental material), the effect we observed in neutrophils likely occurs at the level of this NF-κB cytosolic inhibitor.

Monocytes harbor mature caspase-1, and therefore, a second signal is not required to induce caspase-1 cleavage and activation to convert pro-IL-1β into mature IL-1β (72). Similarly, human neutrophils also harbor cleaved caspase-1 in their cytosol, plasma membrane, and secretory vesicles (47). Indeed, we observed cleaved caspase-1 in unstimulated neutrophils and LPS-treated neutrophils, both of which were reduced by *T. gondii* infection. Interestingly, however, the FLICA assay indicated that the cleaved caspase-1 in unstimulated neutrophils was not enzymatically active. In contrast, treatment with LPS induced high levels of caspase-1 activity, which was impaired by *T. gondii* infection. Thus, our data indicate that there are likely two mechanisms by which *T. gondii* infection modulates IL-1β production in primary human neutrophils: the first is the reduction of LPS-induced NF-κB activation, resulting in reduced *IL-1β* and *NLRP3* transcripts, and the second is the inhibition of caspase-1 cleavage and activation. In addition, in contrast to macrophages and dendritic cells (73), the activation of caspase-1 in neutrophils does not lead to pyroptosis (33–35). Consistent with this, we found that LPS-induced IL-1β release from neutrophils did not induce cell death, and neither did *T. gondii* infection. In neutrophils, this may be due to perturbed expression or function of gasdermin D (74), which is the direct and final executor of pyroptosis in macrophages (75). A disabled pathway of neutrophil pyroptosis may be beneficial to the host, since early pyroptosis may prevent other antipathogenic effector functions.

The differential IL-1β response of monocytes and neutrophils to *T. gondii* infection is intriguing. Although the precise mechanism by which *T. gondii* activates the inflammasome in human monocytes remains unknown, we have found that it depends on NLRP3, ASC, caspase-1, and K^+^ efflux and appears to be “classical” inflammasome activation (26, 39). We have also demonstrated a role for the type II parasite-secreted GRA15 protein in NF-κB activation and *IL-1β* transcription in monocytes (26, 39). Interestingly, both type I and II *T. gondii* inhibit LPS-induced IL-1β release from human neutrophils (data not shown). Although IL-1β regulation is not fully understood in neutrophils, it is curious that *T. gondii* infection and GRA15 secretion in neutrophils do not lead to the same outcome as in monocytes. In addition, there are differences in the mechanisms of inflammasome assembly and activation between monocytes and neutrophils. Neutrophils, like macrophages, express significantly lower levels of NLRP3 than monocytes (45). As a result, monocytes may be more readily stimulated to produce IL-1β during *T. gondii* infection compared to neutrophils and macrophages.

Collectively, the current work provides evidence of a novel pathway by which *T. gondii* manipulates neutrophils, disarming innate immunity by limiting the production of IL-1β during infection, and potentially promoting survival of the parasite.

## MATERIALS AND METHODS

### Isolation of primary human neutrophils.

Human peripheral blood samples from healthy donors were provided by the Institute for Clinical and Translational Science (ICTS) at the University of California, Irvine, in accordance with guidelines and approval of the Institutional Review Board. Blood was mixed with 3% dextran (Sigma-Aldrich) in phosphate-buffered saline (PBS) for 20 min. The top layer containing leukocytes was transferred to a fresh tube, and the cells were underlaid with 15 ml of Ficoll-Paque Plus (GE Healthcare) and centrifuged at 300 × *g* for 20 min. The underlying pellet containing neutrophils and red blood cells (RBC) was suspended in 1× RBC lysis buffer (eBioscience) and incubated for 10 min (35). Neutrophils were washed in PBS and suspended in RPMI 1640 (HyClone) supplemented with 10% heat-inactivated fetal bovine serum (FBS) (Omega Scientific), 2 mM l-glutamine (Corning), 100 U ml^−1^ penicillin, and 100 µg ml^−1^ streptomycin (HyClone) (R-10%). The purity of the isolated population was assessed by flow cytometry and May-Grünwald-Giemsa stain. Isolated human neutrophils were used immediately for experimentation.

### Parasite culture and neutrophil infections.

Type I *T. gondii* (RH) tachyzoites constitutively expressing green fluorescent protein (GFP) or tdTomato were maintained as previously described (76). Syringe-lysed parasites were passed through a 5.0 µm filter unit to remove host cell debris, and neutrophils were infected at a multiplicity of infection (MOI) of 2. Mock-infected controls were samples in which an equal volume of medium without parasites was added to the cells.

### Stimulators and inhibitors.

Ultrapure lipopolysaccharide (LPS) (List Biological) was used at 500 ng ml^−1^ for 30 min or 16 h, and ATP (Sigma-Aldrich) was used at 5 mM to stimulate IL-1β (35, 38). ATP was added with LPS for 30 min or added 3 h after LPS when the cells were stimulated for 16 h.

The caspase-1 inhibitor *N*-acetyl-l-tyrosyl-l-valyl-*N*-[(1*S*)-1-(carboxymethyl)-3-chloro-2-oxo-propyl]-l-alaninamide (Ac-YVAD-CMK) (Cayman Chemical) (42) and the proteasome inhibitor MG-132 (Tocris) (54) were dissolved in dimethyl sulfoxide (DMSO) (Thermo Fisher Scientific). Neutrophils were pretreated with YVAD at 100 µM for 30 min, with MG-132 at 50 µM for 1 h, or with the equivalent volume of DMSO for a vehicle control, and infected and/or stimulated as described above.

*T. gondii* tachyzoites were heat killed by boiling at 100°C for 15 min or pretreated with 5 µM of mycalolide B (Enzo Life Sciences) (44) for 10 min at room temperature and washed twice. For the vehicle control, parasites were treated with an equivalent volume of DMSO.

### Flow cytometry.

Cells were stained as previously described (39) with anti-CD66b antibody conjugated to PerCP-Cy5.5 (anti-CD66b-PerCP-Cy5.5) (G10F5), anti-CD11b antibody conjugated to phycoerythrin (PE) (anti-CD11b-PE) (ICRF44), anti-CD14-PE (HCD14), anti-CD3-PE (SK7), anti-CD20-PE-Cy7 (2H7), anti-CD56 conjugated to allophycocyanin (APC) (anti-CD56-APC) (HCD56) or fluorophore-conjugated isotype controls (all from BioLegend). Cell viability and infection efficiency were also determined in every experiment, as previously described (39). Events were acquired on a FACSCalibur flow cytometer with CellQuest software (BD Bioscience) and analyzed using FlowJo software (TreeStar).

### Enzyme-linked immunosorbent assay (ELISA).

IL-1β released into the supernatant by 10^6^ neutrophils was measured using the ELISA MAX Deluxe Set (BioLegend), according to the manufacturer’s instructions.

### Quantitative real-time PCR.

Total RNA was harvested from 10^7^ neutrophils using the RNeasy kit (Qiagen) and treated with DNase I (Invitrogen). cDNA was generated using SuperScript III first-strand synthesis kit (Life Technologies), according to the manufacturer’s instructions. Previously published primers for *IL-1β*, *NLRP3*, and *GAPDH* (26) were used. Quantitative real-time PCR (Q-PCR) was performed as previously described (77), and data were analyzed using the threshold cycle (ΔΔ*C*_*T*_) method (78). The values obtained for IL-1β and NLRP3 were normalized to those of GAPDH, and the data were expressed as a ratio of mRNA levels.

### Western blotting.

A total of 10^6^ neutrophils were lysed and analyzed by Western blotting as previously described (26) using the following antibodies: anti-IL-1β (3ZD; National Cancer Institute Biological Resources), anti-NLRP3 (D2P5E; Cell Signaling), anti-caspase-1 (Cell Signaling), anti-NF-κB p65 (D14E12; Cell Signaling), anti-phospho-NF-κB p65 (Ser536) (93H1; Cell Signaling), anti-IκBα (L35A5; Cell Signaling), anti-MyD88 (D80F5; Cell Signaling), anti-TRAF6 (D21G3; Cell Signaling), anti-IKKα (Cell Signaling), anti-IKKβ (2C8; Cell Signaling), anti-phospho-IKKα/β (Ser176/180) (16A6; Cell Signaling), anti-CREB1 (48H2; Cell Signaling), anti-phospho-CREB (Ser133) (87G3; Cell Signaling), anti-C/EBPβ (Cell Signaling), anti-phospho-C/EBPβ (Thr235) (Cell Signaling), or anti-β-actin (AC-15; Sigma-Aldrich). Peroxidase-conjugated secondary antibodies were used (BioLegend). Membranes were developed using ECL (Thermo Scientific) and detected using a Nikon camera as previously described (79). Quantification analysis of blots was performed using ImageJ, and β-actin was used as a loading control. The results for samples were expressed as a percentage of the value for the positive control (LPS or LPS+ATP group).

### Caspase-1 activation assay.

Active caspase-1 was quantified by using a FAM-FLICA (fluorescent labeled inhibitor of caspase) detection kit (FAM-YVAD-FMK; ImmunoChemistry Technologies) according to the manufacturer’s instructions. Cell viability was assessed by using Zombie violet (BioLegend). Events were acquired on an imaging flow cytometer (Amnis ImageStream Mark II) and analyzed using IDEAS software.

### Bright field and immunofluorescence microscopy.

Cytospins (Shandon CytoSpin cytocentrifuge) were stained with May-Grünwald-Giemsa solution and analyzed using a Cytation 5 cell imaging multimode reader (BioTek) with a 40× objective.

For immunofluorescence microscopy, neutrophils were settled onto coverslips coated with poly-l-lysine for 1 h at 37°C. The cells were infected for 30 min or 3 h, and the cells were fixed with 4% PFA (Electron Microscopy Sciences). Neutrophils were permeabilized with 0.5% Triton X-100 (Fisher Scientific), blocked with 5% normal goat serum (Southern Biotech), and stained overnight with an anti-myeloperoxidase (anti-MPO) (N4C7[989B]; BioLegend) primary antibody. An Alexa Fluor 594 (AF594)-conjugated secondary antibody (Life Technologies) was used. Coverslips were mounted onto glass slides using ProLong Diamond antifade mountant with DAPI (Life Technologies). Images were acquired using a Nikon Eclipse Ti inverted microscope with a 60× objective and NIS-Elements acquisition software (Nikon Instruments).

### Statistics.

Statistical analyses were performed using GraphPad Instat software. Analysis of variance (ANOVA) followed by Tukey’s or Bonferroni’s test, as indicated, were used for comparison between means. Differences were considered significant when the *P* value was <0.05.
