# Supplementary figures and images for: Evasion of Human Neutrophil-Mediated Host Defense during Toxoplasma gondii Infection
Source: mBio. 2018 Feb 13;9(1):e02027-17. doi: 10.1128/mBio.02027-17 (PMC5821086; doi:10.1128/mBio.02027-17)

Figure S1

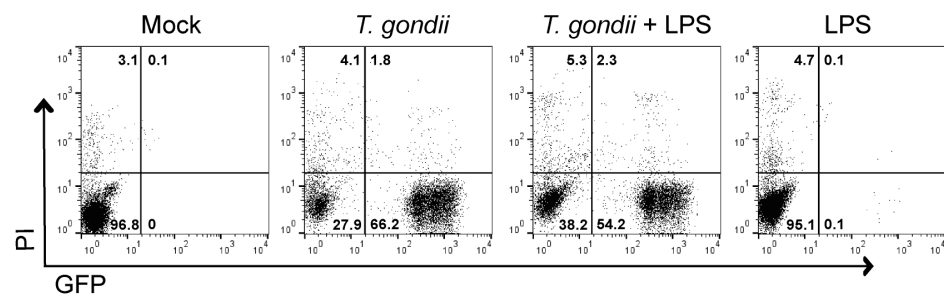

Supplement: FIG S1 [file mbo001183713sf1.pdf]

Figure S2

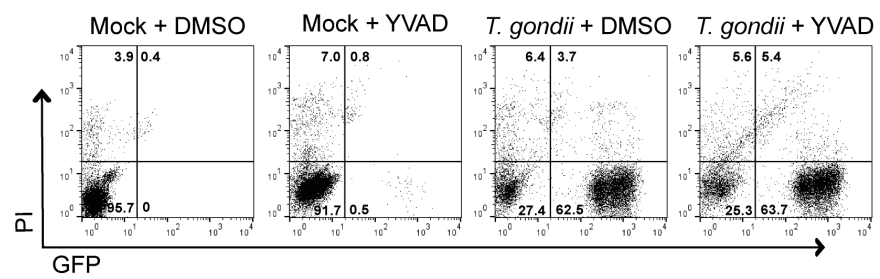

Supplement: FIG S2 [file mbo001183713sf2.pdf]

Figure S3

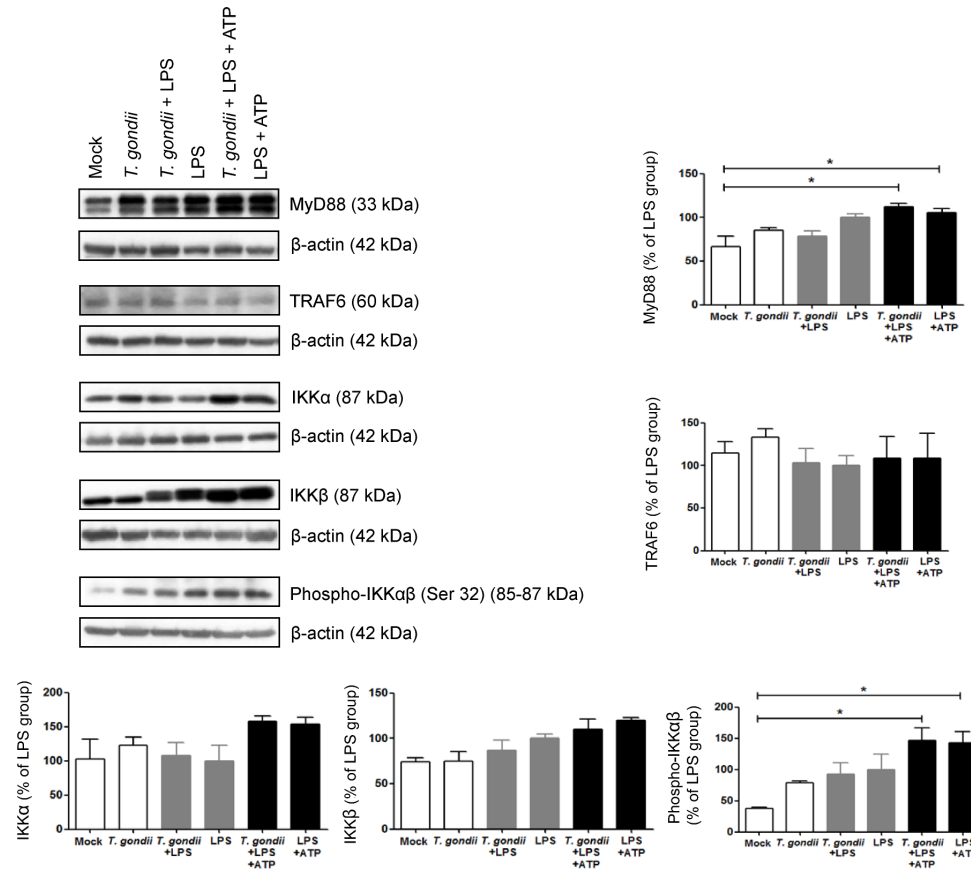

Supplement: FIG S3 [file mbo001183713sf3.pdf]

Figure S4

**A**

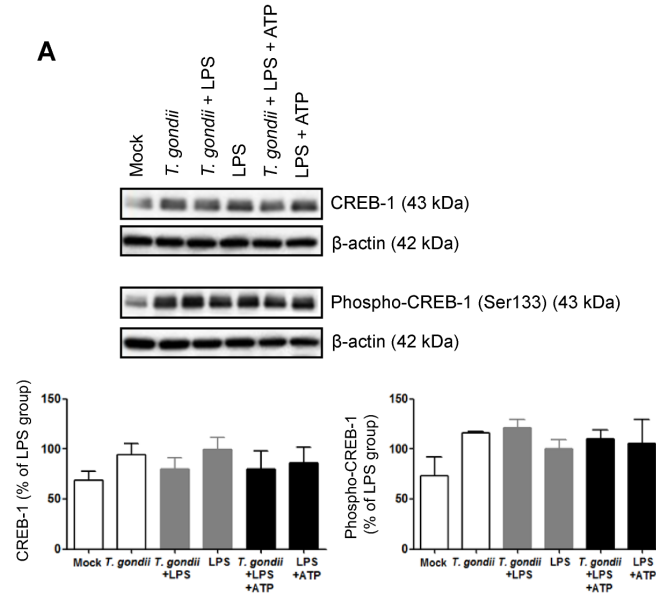

**B**

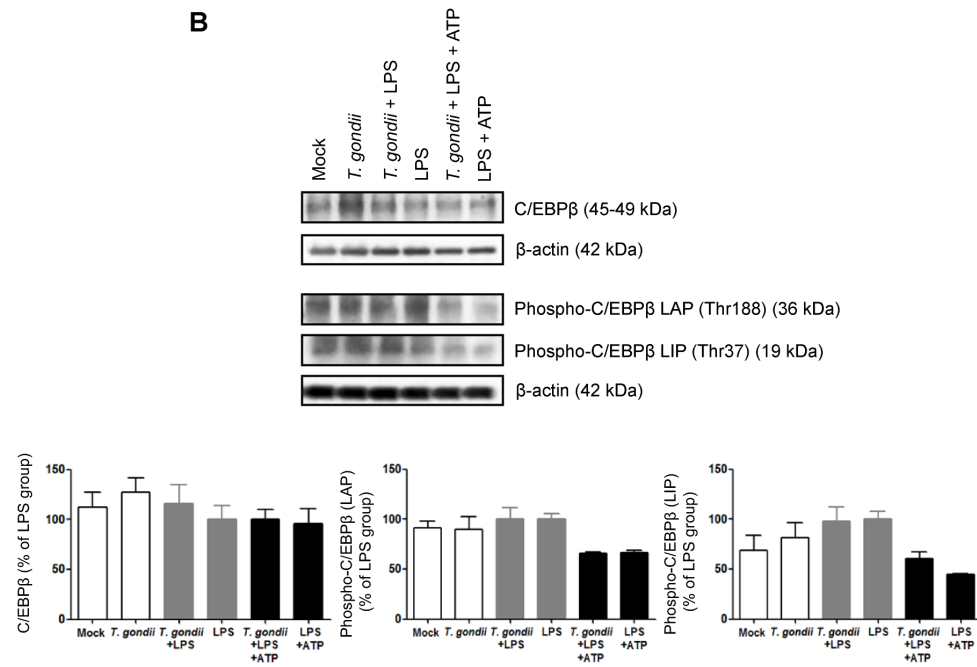

Supplement: FIG S4 [file mbo001183713sf4.pdf]
